# ACAT2 may be a novel predictive biomarker and therapeutic target in lung adenocarcinoma

**DOI:** 10.1002/cnr2.1956

**Published:** 2024-01-11

**Authors:** Zhongchao Wang, Zhugen Cao, Zhaoxia Dai

**Affiliations:** ^1^ The Second Hospital Dalian Medical University Dalian China; ^2^ Xinyi People's Hospital Xinyi China; ^3^ Suqian First People's Hospital Suqian China

**Keywords:** ACAT2, lung adenocarcinoma, prognosis

## Abstract

**Background:**

Acyl‐coenzyme A cholesterol acyltransferase (ACAT) is a membrane‐binding enzyme localized in the endoplasmic reticulum. ACAT2 can promote the development of colon cancer, but its efficacy in lung adenocarcinoma (LUAD) remains uncertain.

**Method:**

ACAT2 expression was performed by using the TIMER2.0 database. The GEPIA database was utilized to analyze the correlation between ACAT2 expression and pathological stage of the tumor. Clinical prognosis was assessed through the Kaplan–Meier analysis. The CancerSEA database was employed to scrutinize the correlations between the ACAT2 expression and the functional status of various tumors, which were subsequently visualized as a heatmap. Furthermore, molecular interaction network analysis was performed by the STRING tool.

**Results:**

High ACAT2 expression was associated with a poor DFS and OS in LUAD patients. Cox regression analysis indicated that the poor outcomes may be related to tumor stage, nodal stage, distant metastatic stage. ACAT2 was found to play a crucial role in various biological processes, including the cell cycle, DNA repair, DNA damage response, and proliferation. Enrichment pathway analysis revealed four ACAT2 related genes, ACOX1, EHHADH, OXCT1, and DLAT.

**Conclusion:**

Our study showed that ACAT2 was upregulated in LUAD, and had a worse survival. ACAT2 could be a novel predictive biomarker and therapeutic target in LUAD.

## 1 INTRODUCTION

In China, lung malignancies are one of the most usual and lethal cancers. Nonsmall cell lung cancer (NSCLC) accounts for about 85%, and is the most common type.^1^ The most prevalent pathological type is lung adenocarcinoma (LUAD).^2^ Primary treatment approaches include surgery, immunotherapy, chemotherapy, radiotherapy and targeted therapy. Early‐detected lung carcinoma has a better prognosis after surgery. However, most lung cancer patients, who lose the opportunity of surgical treatment, are diagnosed in the middle or late stages. The overall survival (OS) rate of 5‐year is low (~18.1%).^3^ Lung cancer is asymptomatic at an earlier preclinical stage. As a result, early diagnosis is difficult. Therefore, it is highly warranted to explore novel non‐invasive diagnostic molecular biomarkers for the detection the early stage lung carcinoma, prognosis assessment and an individualized treatment planning.

Acyl‐coenzyme A cholesterol acyltransferase (ACAT) is a membrane‐binding enzyme localized in the endoplasmic reticulum. The formation of the bycholesteryl ester is obtained by ACAT catalyzing the long chain fatty acid acyl‐CoA and cholesterol.^4^ Excess cholesterol esters are stored in the cells as lipid droplets. ACAT1 and ACAT2 are isoforms of the ACAT family. ACAT1 is ubiquitously expressed in normal human tissues and generates sterol esters that are integrated into cellular lipid droplets.^5^ ACAT2 provides the cholesteryl esters for lipoprotein assembly, which is principally expressed in the fetal liver and intestines.^6^ In clear cell renal cell carcinoma, the decreased expression of ACAT2 indicates a poor prognosis.^7^ A recent study shows that ACAT2 is overexpressed in tissues and cell lines of colon cancer (CRC). High expression of ACAT2 is associated with rapid malignant progression and poor patient outcomes. In vitro, downregulation of ACAT2 inhibited the growth of CRC cells.^8^ ACAT2 was identified frequent promoter hypermethylation‐mediated inactivation in primary LUAD from smokers and never smokers. 6‐phosphogluconate dehydrogenase (6PGD) activity, an important enzyme for cancer pathogenesis and tumor development, was increased in many cancers. Knockdown of ACAT2 resulted in decreased lysine acetylation of 6PGD, then blunted tumor growth. Nonetheless, the relationship between ACAT2 expression and LUAD prognosis and their molecular mechanisms is still unclear.

In our research, we aim to examine the expression of ACAT2 in pan‐cancer and LUAD tissues, and to explore the prognostic significance of ACAT2 in LUAD patients.

## 2 MATERIALS AND METHODS

### 2.1 Expression level of ACAT2 in pan‐cancer and LUAD patients

To explore the expression of ACAT2 mRNA between normal tissues and tumor in pan‐cancer and LUAD tissues, we used The Cancer Genome Atlas (TCGA) program and Xiantao bioinformatics tool (https://www.xiantao.love/products). ACAT2 was input in the DiffExp module. We selected the TCGA module and entered the gene symbol ACAT2. Immunohistochemical analysis was performed by the human protein atlas (https://www.proteinatlas.org/). We selected relevant normal tissue names and tumor tissue names.

### 2.2 Prognostic significance of ACAT2 in LUAD patients

In order to explore the prognostic significance of ACAT2, correlations between expression of ACAT2 and functional status of tumor were analyzed by the gene expression profiling interactive analysis (GEPIA database; http://gepia.cancer-pku.cn/)，which includes 8587 normal tissues and 9736 tumor tissues. The cutoff value was the median, 95% confidence interval (CI) was the dotted line, the horizontal axis unit was month. To compare the differences of disease‐free survival (DFS) and OS, Kaplan–Meier (K–M) curves were plotted (https://kmplot.com/analysis/). A Cox proportional hazard model was used for univariate and multivariate survival analysis by Xiantao bioinformatics tool. Independent prognostic factor analysis was performed using Xiantao bioinformatics toolbox. According to the difference of clinical features, the parameters are as follows: hazard ratio was “yes”，cutoff value was “median”, and 95%CI was “yes”.

### 2.3 Correlations between the ACAT2 expression and tumor functional status

CancerSEA database analyzed correlations between the expression of ACAT2 and functional status of different tumors displayed as a heatmap (http://biocc.hrbmu.edu.cn/CancerSEA/). There were 14 functional statuses in the database, namely apoptosis, angiogenesis, DNA damage, invasion, cell cycle, EMT, differentiation, DNA repair, proliferation, inflammation, hypoxia, metastasis, quiescence, and stemness. The expression profiles of ACAT2 were displayed at the single‐cell level using the T‐SNE diagram. We entered the web input ACAT2 gene, and searched.

### 2.4 ACAT2‐related genes enrichment analysis

The molecule interaction network analysis performed by the STRING tool (https://cn.string-db.org/). We input ACAT2, and selected auto‐detect. Organism was homo sapiens. Pearson's coefficient was utilized to analyze ACAT2 and the selected genes using the Xiantao bioinformatics tool. Gene ontology (GO) and the Kyoto Encyclopedia of Genes and Genomes (KEGG) enrichment assay of ACAT2 related genes were carried out in the Xiantao bioinformatics tool.

### 2.5 Statistical analyses

In the TIMER2.0 database, the Wilcoxon test was used for statistical significance. For the comparison of tumor and normal samples, the ANOVA method was used in GEPIA2 database. The data were represented by mean ± SD. Correlations between ACAT2 and its associated genes were analyzed using Spearman's correlation coefficient. The OS and DFS analyses in LUAD patients were used for the Kaplan–Meier curves and log‐rank tests. GraphPad Prism 7.0 version software and SPSS 17.0 version software were used to analyze the data. Values of *p* < .05 were considered to be statistically signifcant differences.

## 3 RESULTS

### 3.1 Abnormal expression with ACAT2 in pan‐cancer and LUAD patients

**FIGURE 1:**
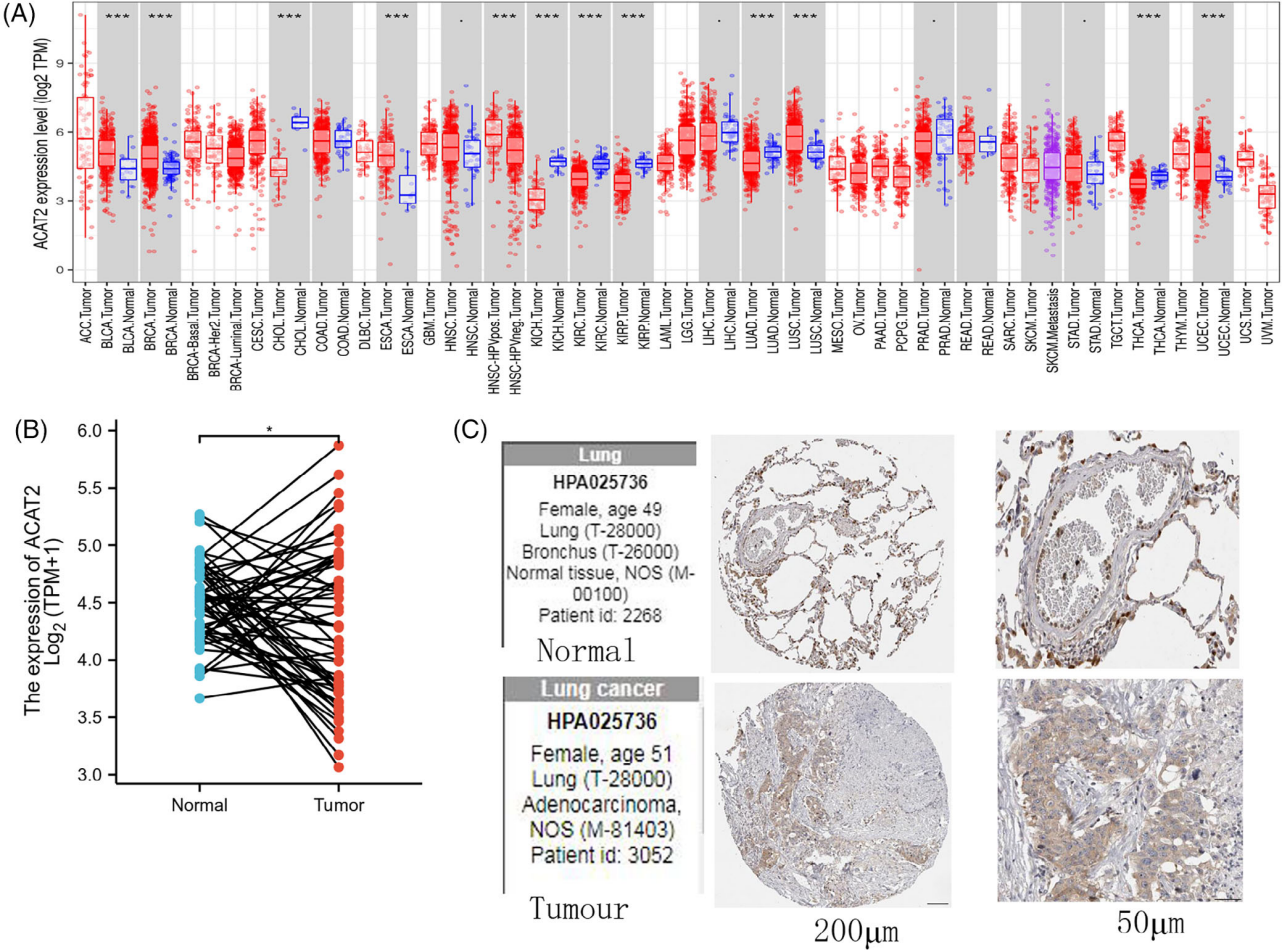
Abnormal ACAT2 expression in pan‐cancer and LUAD patients. (A) TIMER2 database was performed to detect ACAT2 mRNA level. (B) The unpaired samples examined ACAT2 mRNA expression level in LUAD and normal group. (C) Immunohistochemistry analysis was performed in normal and tumor tissues (Normal:HPA025736, female, age 49, patient id 2268. Tumor: HPA 025736, female, age 51, patient id 3052). *p*‐value: ns *p* > .05, * *p* < .05, ** *p* < .01, and *** *p* < .001.

In our study, to compare with the ACAT2 mRNA expression levels in tumor and normal tissues, The Cancer Genome Atlas (TCGA) program and Xiantao bioinformatics tool were performed (Figure 1A). The ACAT2 mRNA levels were overexpressed in pan‐cancer. For instance, bladder urothelial carcinoma (BLCA), esophageal squamous cell carcinoma (ESCA), breast invasive carcinoma (BRCA), kidney renal clear cell carcinoma (KIRC), cholangiocarcinoma (CHOL), LUAD, head and neck squamous cell carcinoma‐human papillomavirus positive (HNSC‐HPVpos), lung squamous cell carcinoma (LUSC), head and neck squamous cell carcinoma‐human papillomavirus negative (HNSC‐HPVneg), kidney renal papillary cell carcinoma (KIRP), uterine corpus endometrial carcinoma (UCEC), thyroid carcinoma (THCA). Conversely, ACAT2 manifested low expression in stomach adenocarcinoma (STAD), head‐and‐neck squamous cell carcinoma (HNSC), prostate adenocarcinoma (PRAD), and liver hepatocellular carcinoma (LIHC).

To further explore the differential expression of ACAT2 in LUAD patients, the expression of ACAT2 mRNA was performed by Xiantao bioinformatics toolbox. The expression level of ACAT2 in LUAD tissues was higher than normal tissues (Figure 1B). To test whether ACAT2 was also upregulated in tumor tissues, immunohistochemistry analysis was performed in normal and tumor tissues. Tumor tissues had significantly higher expression levels of ACAT2, when compared to normal adjacent tissues. (Figure 1C).

### 3.2 Prognostic analysis of ACAT2 in LUAD patients

**FIGURE 2:**
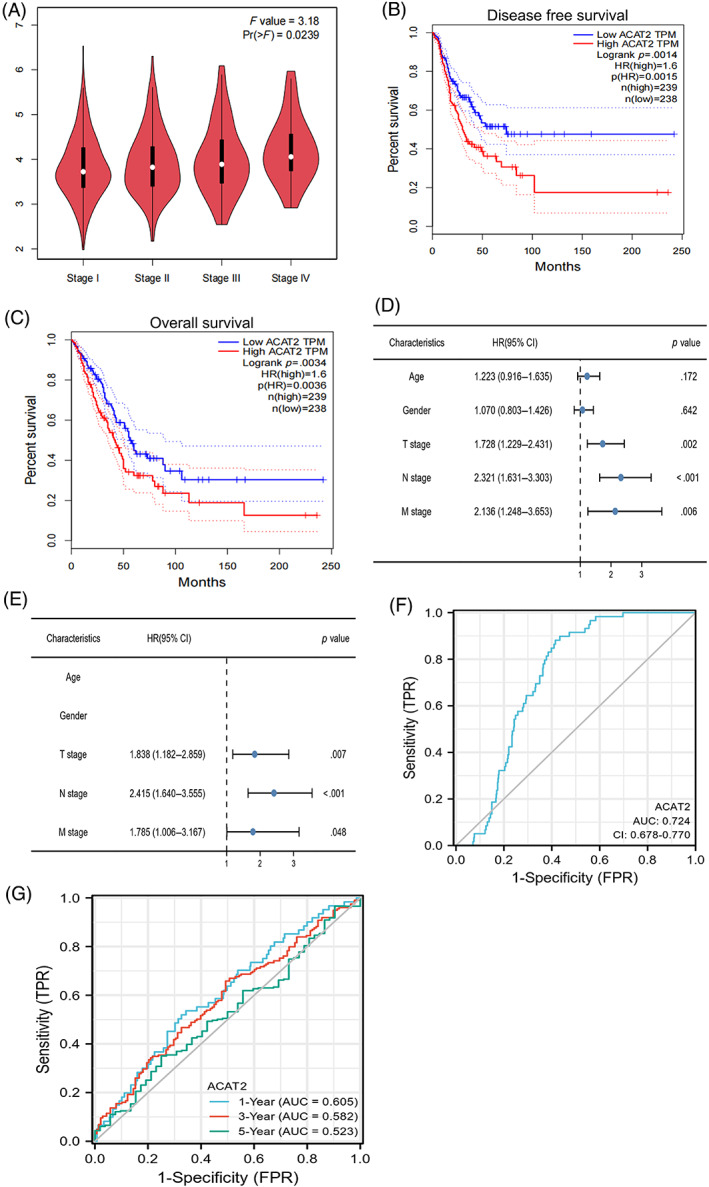
Prognostic analysis of ACAT2 in LUAD patients. (A) GEPIA2 showed relations between the expression of ACAT2 and pathological stage of tumor. (B) DFS survival curves between high expression ACAT2 and low expression ACAT2 groups. (C) OS survival curves between high expression ACAT2 and low expression ACAT2 groups. (D) Independent prognostic factors were performed by univariate COX regression analysis. (E) Independent prognostic factors were performed by multivariable COX regression analysis. (F) The ACAT2 ROC curve. (G) OS of the area under the ROC curve was analyzed to predict the survival at 1‐, 3‐, and 5‐year periods.

We used the GEPIA2 tool to explore the relationship between the expression of ACAT2 and tumor pathological stage. There was a significant correlation between the expression of ACAT2 and tumor staging pathological stage (Figure 2A). Furthermore, compared with the high expression group, Kaplan–Meier analysis also demonstrated that the DFS and OS of the low expression lever were better (Figure 2B,C). A Cox proportional hazard model was used for univariate and multivariate survival analysis. As a result, univariate and multivariate Cox regression analysis indicated that the poor outcomes may be related to the tumor stage, nodal stage, distant metastatic stage (Figure 2D,E). The predictive effect of ROC profile exceeded other clinical factors (sex, age, KPS, pathological stage, and so on; Figure 2F). Model for predicting the sensitivity and specificity of patients was assessed by time‐dependent ROC curves. During 1‐, 3‐, and 5‐year periods, the OS scores of the area under the ROC curve were 0.605, 0.582, and 0.523, respectively (Figure 2G).

### 3.3 Correlation between the expression of ACAT2 and functional status of different tumors

**FIGURE 3:**
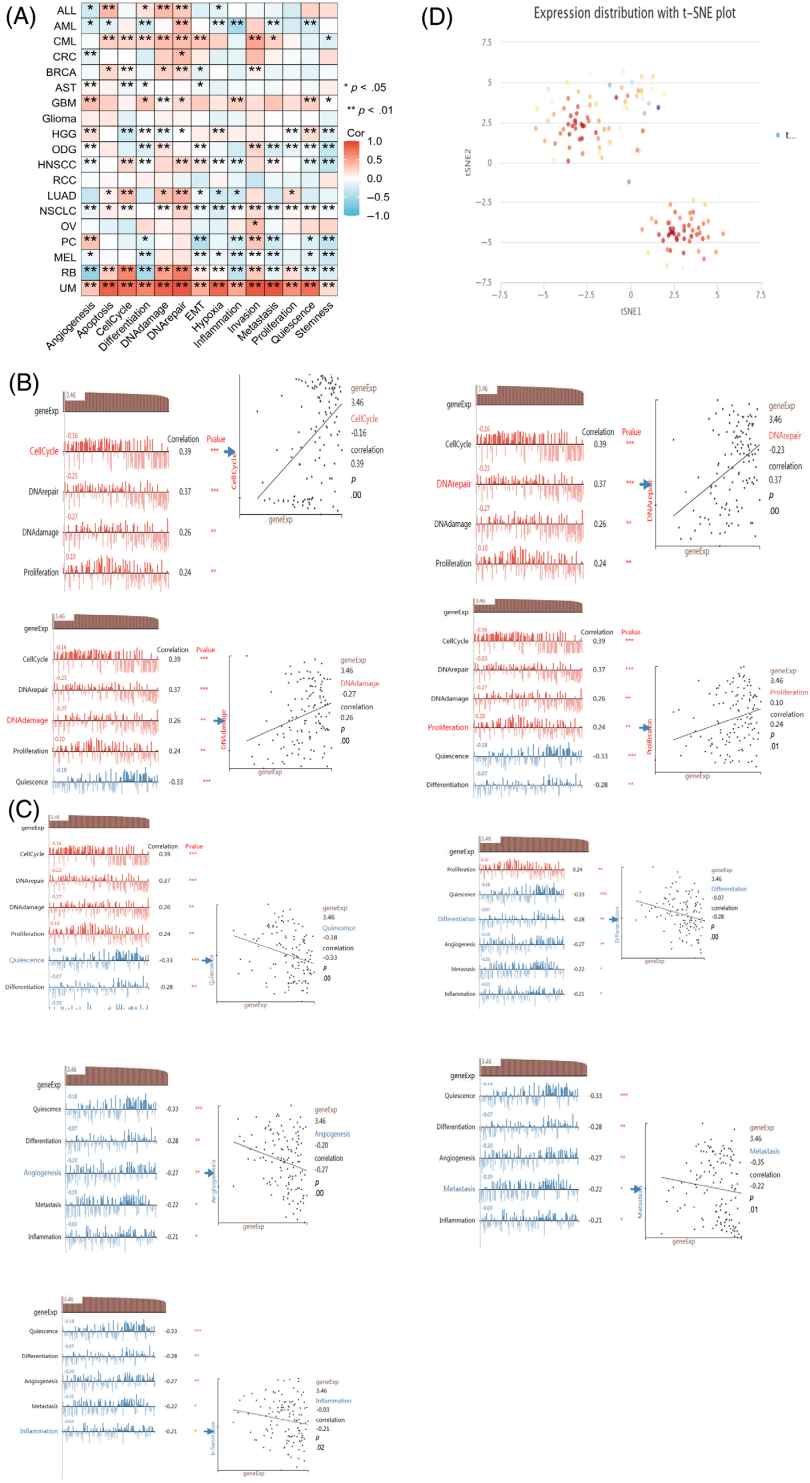
Correlation between the ACAT2 expression and different tumor functional status. (A) CancerSEA database displayed the correlation between functional status of different tumor and the expression of ACAT2. (B, C). Different functional status of the ACAT2‐related expression. (D) The T‐SNE diagram for LUAD at single‐cell levels showed ACAT2 expression profiles. *p*‐value: ns. *p* > .05, * *p* < .05, ** *p* < .01, and *** *p* < .001.

Single cell sequencing technology was used to study cancer heterogeneity. CancerSEA database at the single cell level was utilized to detect relations between ACAT2 expression and its correlation with the tumor functional status in different cancers. There was high correlation between the expression of ACAT2 and fourteen tumor functional statuses in the heatmap (Figure 3A). ACAT2 showed a significantly and positively correlation with cell cycle (*r* = .39, *p* = .00), DNA repair (*r* = .37, *p* = .00), DNA damage (*r* = .26, *p* = .00), and proliferation (*r* = .24, *p* = .01; Figure 3B). Simultaneously, there were strong negative relations between ACAT2 and quiescence (*r* = −.33, *p* = .00), differentiation (*r* = −.28, *p* = .00), angiogenesis (*r* = −.27, *p* = .00), metastasis(*r* = −.22, *p* = .01), inflammation (*r* = −.21, *p* = .02; Figure 3C). The T‐SNE diagram for LUAD at the single‐cell levels showed ACAT2 expression profiles (Figure 3D). The above evidence revealed that ACAT2 might play a vital part in the biological processes involved in the progression of cancer.

### 3.4 Analysis of the ACAT2‐correlated co‐expression network and the enrichment pathway

**FIGURE 4:**
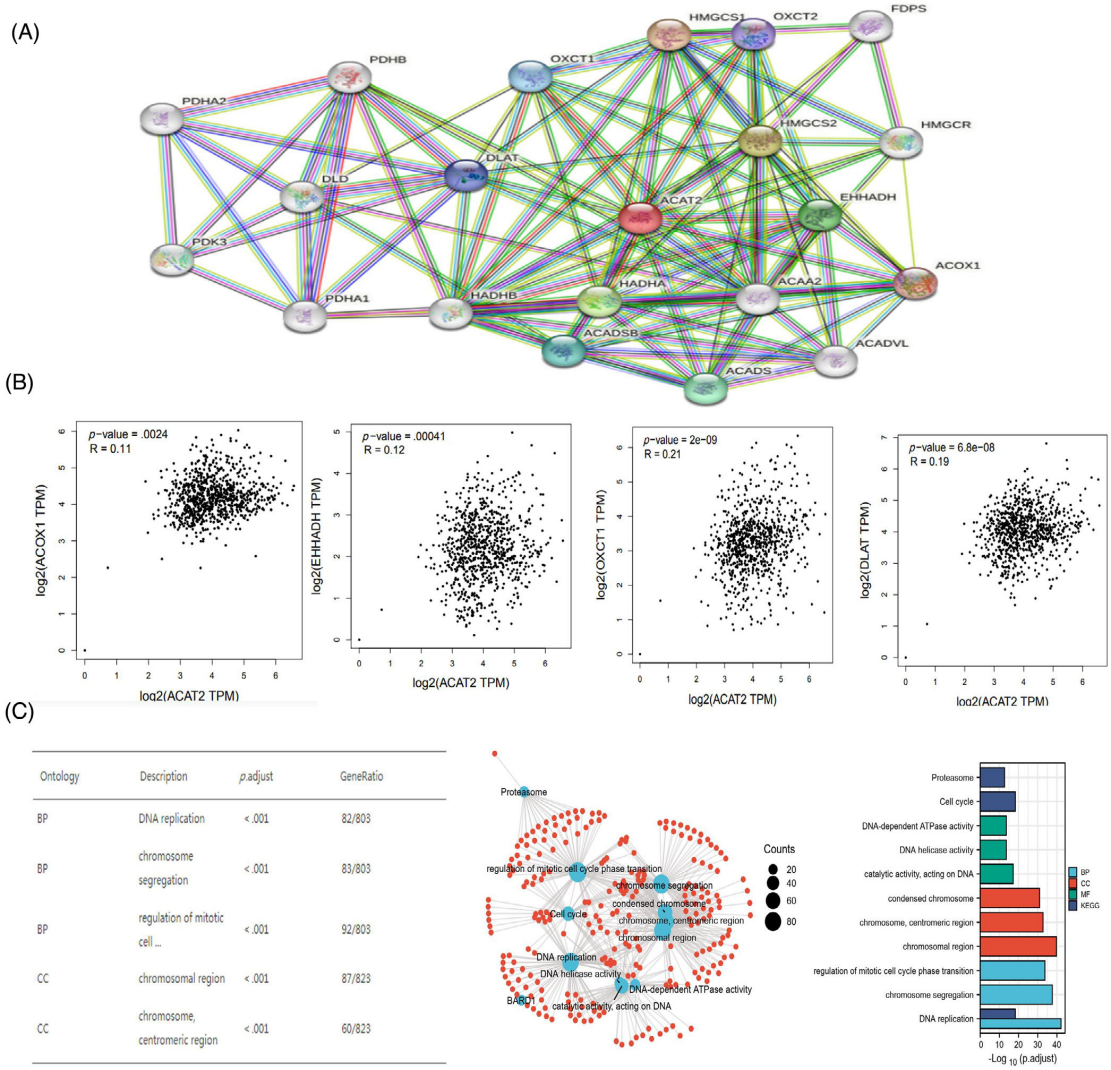
Analysis of the ACAT2‐correlated co‐expression network and the enrichment pathway. (A) STRING showed ACAT2‐binding molecules. (B) GEPIA2 was used to analyze 4 of ACAT2‐correlated genes. (C) Chart of the GO and KEGG enriched pathways.

To further elucidate the molecular mechanism of ACAT2, ACAT2‐related molecular networks were constructed by the STRING tool. ACAT2‐binding molecules were acquired, as shown in (Figure 4A). The top four ACAT2‐correlated genes were analyzed by GEPIA2 database. The expression of ACAT2 positively correlated with acyl‐CoA oxidase 1 (ACOX1, *r* = .11, *p* = .0024), enoyl‐CoA, hydratase/3‐hydroxyacyl CoA dehydrogenase (EHHADH, *r* = .12, *p* = .00041), 3‐oxoacid CoA‐transferase 1 (OXCT1, *r* = .21, *p* = 2e‐09), dihydrolipoamide S‐acetyltransferase (DLAT, *r* = .19, *p* = 6.8e‐08; Figure 4B). GO and KEGG pathway analysis exposed ACAT2‐related biological processes and cellular components (Figure 4C). DNA replication, chromosome segregation, regulation of mitotic cell cycle phase transition, chromosomal and centromeric region, condensed chromosome, chromosome, activity of catalytic, activity of DNA helicase and DNA‐dependent ATPase, cell cycle were some of the examples of ACAT2‐related biological processes and cellular components.

## 4 DISCUSSION

ACAT plays pivotal parts in the cholesterol metabolism pathway. The ACAT family includes ACAT1 and ACAT2 two isoforms. ACAT1 participates in the metabolism of ketogenesis, and is localized in mitochondrial matrix. ACAT1 is widely expressed in multiple tumor types, while ACAT2 is predominantly distributed in some organs, like small intestine and liver.^9^ Its mutations have been reported to contribute to a wide variety of diseases.^10^ Insulin indirectly drives the development of CRC by increasing in the activity of ACAT1, and the above results suggest that ACAT1 could be an effective anti‐cancer target for CRC.^11^ The overexpression of ACAT1 is associated with neoplastic progression and poor prognosis in human high‐grade prostate, pancreatic and breast cancers.^12^, ^13^, ^14^

ACAT2 is involved in the production of cytosolic acetoacetyl‐CoA. However, very little is known about other mechanisms. Recently, a new study has shown that the inflammatory responses or liver stress resulting from ACAT2 overexpression also inhibit the metabolic pathways of glycolytic, TG synthesis, mitochondrial‐related and ketone body metabolism, but the upregulated genes participate in the metabolism of cholesterol, particularly the biosynthesis pathway of bile acid.^15^ ACAT2 is significantly upregulated, and promotes cancer cell growth and progression of CRC.^8^ However, ACAT2 is downregulated in KIRC.^7^

In our results, a pan‐cancer analysis found that ACAT2 was overexpressed in CHOL, BRCA, ESCA, BLCA, HNSC‐HPVpos, HNSC‐HPVneg, UCEC, KIRC, LUAD, KIRP, LUAD, THCA, and LUSC. While low expression of ACAT2 was observed in HNSC, LIHC, PRAD, and STAD. The findings revealed that ACAT2 might play distinct parts in the course of tumourigenesis. In addition, high expression of ACAT2 in LUAD had a poor DFS and OS, compared to low expression of ACAT2. Univariate and multivariate cox regression analysis indicated that poor outcomes might be related to the tumor stage, nodal stage, distant metastatic stage. OS of the area under the ROC curve was 0.605, 0.582, and 0.523 for 1‐, 3‐, and 5‐year periods, respectively. This revealed that ACAT2 might be a predictive biomarker of unfavorable outcomes.

GO enrichment analysis elucidated that ACAT2 participated in the biological processes of cell cycle, DNA repair, DNA damage, and proliferation. These processes were indispensable for maintaining diverse physiological events. If any of these processes were deregulated, cancer or other diseases might occur. Enrichment pathway analysis showed four ACAT2‐correlated genes, acyl‐CoA oxidase‐1 (ACOX1), enoyl‐CoA‐hydratase/3‐hydroxyacyl CoA dehydrogenase (EHHADH), 3‐oxoacid CoA‐transferase 1 (OXCT1), dihydrolipoamide s‐acetyltransferase (DLAT). ACOX1 was a highly conserved enzyme that played a key role in lipid metabolism. Inhibition of ACOX1 caused multiple metabolic disorders by improving the metabolism of the reactive oxygen species (ROS) and mitochondrial lipid.^16^ EHHADH participated in fatty acids degradation. A recent study found that EHHADH depletion restrained proliferation, invasion, and migration of cancer cells, and facilitated the sensitivity of bladder cancer (BC) cells to cisplatin in vitro.^17^ OXCT1, an important rate‐limited enzyme, was involved in the metabolism of ketone body and the production of ATP.^18^ Compared with the low OXCT1 expression group, the patients’ relapse‐free survival (RFS) was substantially shorter in high OXCT1 expression group.^19^ DLAT highly expressed in HCC specimens, and high DLAT expression was associated with poor prognosis.^20^

We use several online tools to discuss the ACAT2 expression in LUAD, and expression of ACAT2 is associated with prognosis. Nevertheless, our study has some limitations. First, the database may only cover data for a specific time period or a specific region. The database may not be up to date, or there may be some cases of missing or inconsistent data. All of these factors may have an impact on the study findings. Second, gene set enrichment analysis of ACAT2 have not been explored. Third, it is essential to explore the potential mechanisms of ACAT2 in vivo and in vitro.

## 5 CONCLUSION

In summary, ACAT2 is upregulated in LUAD tissues. Highly expressed ACAT2 has a poor clinical prognosis. However, our results require clinical verification, and we will further investigate the mechanisms of ACAT2 in vivo and in vitro.

## AUTHOR CONTRIBUTIONS

**Zhongchao Wang:** Investigation (equal); writing – original draft (equal). **Zhugen Cao:** Data curation (equal). **Zhaoxia Dai:** Methodology (equal); writing – review and editing (lead).

## CONFLICT OF INTEREST STATEMENT

The authors declare no conflicts of interest.

## ETHICS STATEMENT

Ethical approval for this investigation was obtained from the Research Ethics Committee, the second hospital of Dalian Medical University.

## Data Availability

N/A.
